# Katanin catalyzes microtubule depolymerization independently of tubulin C‐terminal tails

**DOI:** 10.1002/cm.21522

**Published:** 2019-05-02

**Authors:** Liudmila Belonogov, Megan E. Bailey, Madison A. Tyler, Arianna Kazemi, Jennifer L. Ross

**Affiliations:** ^1^ Department of Physics University of Massachusetts Amherst Massachusetts; ^2^ Molecular and Cellular Biology Graduate Program University of Massachusetts Amherst Massachusetts

**Keywords:** AAA ATPase, cytoskeleton, E‐hook, fidgetin, severing enzyme, spastin, subtilisin, taxol, tubulin

## Abstract

Microtubule network remodeling is an essential process for cell development, maintenance, cell division, and motility. Microtubule‐severing enzymes are key players in the remodeling of the microtubule network; however, there are still open questions about their fundamental biochemical and biophysical mechanisms. Here, we explored the ability of the microtubule‐severing enzyme katanin to depolymerize stabilized microtubules. Interestingly, we found that the tubulin C‐terminal tail (CTT), which is required for severing, is not required for katanin‐catalyzed depolymerization. We also found that the depolymerization of microtubules lacking the CTT does not require ATP or katanin's ATPase activity, although the ATP turnover enhanced depolymerization. We also observed that the depolymerization rate depended on the katanin concentration and was best described by a hyperbolic function. Finally, we demonstrate that katanin can bind to filaments that lack the CTT, contrary to previous reports. The results of our work indicate that microtubule depolymerization likely involves a mechanism in which binding, but not enzymatic activity, is required for tubulin dimer removal from the filament ends.

## 1 INTRODUCTION

Microtubule severing enzymes are essential regulators of the microtubule cytoskeleton impacting the intracellular organization during important processes including axon outgrowth, mitosis, cell migration, and plant cell wall organization and deposition. Microtubule severing enzymes remodel cytoskeletal networks by removing microtubules and generating shorter microtubules that can be used as seeds for a variety of diverse purposes (Nakamura, Ehrhardt, & Hashimoto, 2010; Roll‐Mecak & Vale, 2005; Ahmad et al, 1999; Karabay et al, 2004; Srayko, Buster, Bazirgan, McNally, & Mains, 2000; Srayko, O'Toole, Hyman, & Muller‐Reichert, 2006). Recent work has also suggested that severing enzymes might remove individual dimers from the sides of microtubules to allow new dimers to bind within the lattice to strengthen filaments (Aumeier et al., 2016; Schaedel et al., 2015; Vemu et al., 2018).

The current theory for how katanin severs microtubules is that it uses ATP to oligomerize into a hexamer and then disassembles microtubules by threading the carboxy‐terminal tail (CTT) of tubulin through its pore (Hartman et al., 1998; Roll‐Mecak & Vale, 2008; Vale, 2000). After docking on the side of the microtubule, there are two proposed mechanisms for how the dimer is removed. In the “unfoldase” mechanism, the severing enzyme uses its ATPase to pull the CTT through the central pore and unravel the dimer. In the “wedge” mechanism, katanin docks to the microtubule with the CTT but uses its ATPase to wedge between dimers, only holding on by the CTT. It is also possible that both activities could occur simultaneously (Barsegov, Ross, & Dima, 2017; Vale, 2000).

Regardless of the mechanism, it is well known that the CTT of tubulin is essential for severing (Johjima et al., 2015; McNally & Vale, 1993; Vale, 1991). We recently showed in a minimal in vitro system containing no other microtubule or katanin regulatory proteins, katanin severing can be inhibited by the presence of free dimers of tubulin or free CTT peptides (Bailey, Sackett, & Ross, 2015). This process effectively creates a negative feedback loop, where katanin severing microtubules releases free tubulin dimers, increasing the concentration of free tubulin. The katanin then binds to the newly released free tubulin, inhibits its activity on microtubules, and shuts down severing. We found that katanin had a higher affinity for the CTT than for tubulin within the microtubule lattice, because relatively low concentrations of CTT peptides effectively compete with the microtubule for katanin binding (Bailey et al., 2015). The demonstrated preference of katanin for CTTs and prior work showing that subtilisin‐treated microtubules lacking the CTTs of tubulin are safe from severing (Johjima et al., 2015; McNally & Vale, 1993; Vale, 1991) all imply that the CTT is the main and most essential target for katanin severing.

Several groups, including our own, have noted that katanin has the ability to depolymerize microtubules in addition to severing them (Díaz‐Valencia et al., 2011; McNally & Vale, 1993; Zhang et al., 2011). This activity is important when regulating microtubule length, particularly at the cortex in interphase cells and kinetochore fibers in mitosis (Jiang, Bailey, Burke, Ross, & Dima, 2017b; Jiang, Rezabkova, et al., 2017a; McNally, Audhya, Oegema, & McNally, 2006; Zhang et al., 2011; Zhang, Rogers, Buster, & Sharp, 2007). We have shown that katanin can depolymerize taxol‐stabilized microtubules in an ATP‐dependent manner (Díaz‐Valencia et al., 2011; Zhang et al., 2011). We previously proposed two possible mechanisms for the end‐shortening of microtubules: (a) shrinkage due to dissociation of individual tubulin dimers, catalyzed by katanin binding and (b) severing of subdiffraction sized microtubules or multimers broken off and diffusing out of plane, equivalent to katanin severing.

Here, we provide evidence to suggest microtubule shrinkage events catalyzed by katanin activity progress using a mechanism different from that of severing. Specifically, we show that microtubules lacking the CTT can depolymerize, and the depolymerization depends on the binding of katanin. We observe that depolymerization occurs in the absence of ATP, and depolymerization rates are enhanced with ATP. Finally, we directly observed katanin binding to microtubules that lack the CTT. Our results also support a mechanism where the katanin can bind to microtubule ends and act like a wedge to remove dimers.

## 2 RESULTS

### 2.1 Microtubules lacking the CTT lost mass in a Katanin‐dependent manner

**Figure 1:**
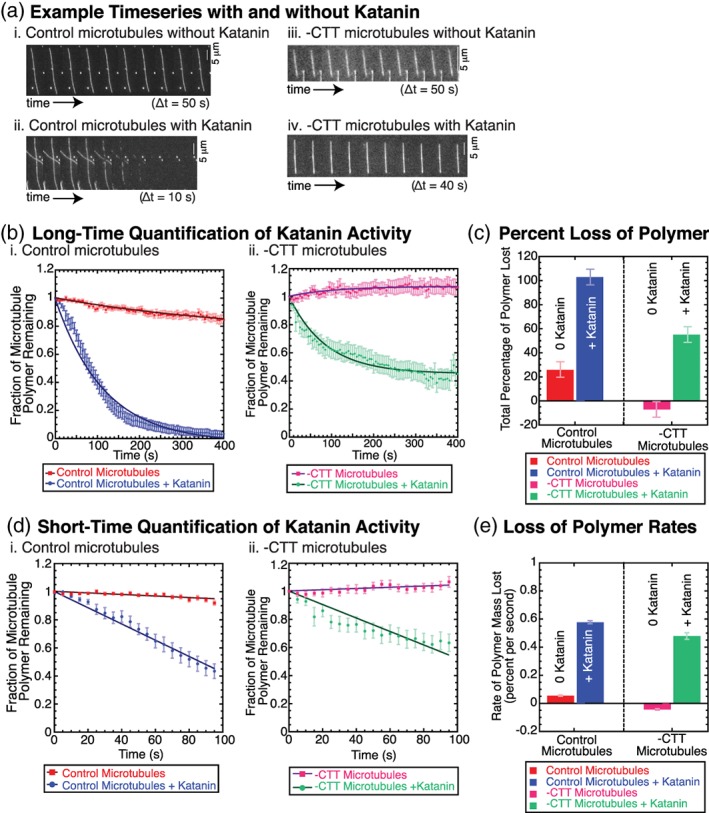
Katanin activity (250 nM) on microtubules results in polymer loss of taxol‐stabilized microtubules with or without the CTT. (a) Example timeseries (i) for control microtubules without katanin (time between frames 50 s), (ii) for control microtubules with katanin (time between frames 10 s), (iii) for ‐CTT microtubules without katanin (time between frames 50 s), and (iv) for ‐CTT microtubules with katanin (time between frames 40 s). Scale bars 5 μm. All microtubules were taxol stabilized, and a single preparation of katanin was used. (b) Long time data: Total loss of polymer over 400 s for (i) control microtubules without katanin (red squares, *N* = 43 microtubules in six chambers) and with katanin (blue circles, *N* = 35 microtubules in seven chambers), (ii) for ‐CTT microtubules without katanin (magenta squares, *N* = 33 microtubules in four chambers) and with katanin (green circles, N = 32 microtubules in six chambers). Data fit (lines through data sets) to Equation (1). Error bars represent standard error of the mean for average over *N* microtubules. (c) The percent of polymer lost from microtubules plotted for control microtubules without katanin (red bar), and control microtubules with katanin (blue bar), ‐CTT microtubules without katanin (magenta bar), and ‐CTT microtubules with katanin (green bar). Error bars represent the minimum uncertainty of the measurement, 7%. (d) Short time data: Total loss of polymer over the first 100 s for (i) control microtubules without katanin (red squares, *N* = 43, six chambers) and with katanin (blue circles, *N* = 35 microtubules in seven chambers), and (ii) ‐CTT microtubules without katanin (magenta squares, *N* = 33 microtubules in four chambers) and with katanin (green circles, *N* = 32 microtubules in six chambers). Data fit (lines through data sets) to Equation (2). Error bars represent standard error of the mean over *N* microtubules. (e) The rate of polymer loss from Equation (2) fits plotted for control microtubules without katanin (red bar), and control microtubules with katanin (blue bar), ‐CTT microtubules without katanin (magenta bar), and ‐CTT microtubules with katanin (green bar). Error bars represent the error in the rate parameter from Equation (2). All fit parameters are reported in Tables S1–S4. CTT, C‐terminal tail [Color figure can be viewed at wileyonlinelibrary.com]

The CTT of tubulin is essential for katanin‐dependent severing, as previously shown (Johjima et al., 2015; McNally & Vale, 1993; Vale, 1991). We used wild‐type *Xenopus laevis* katanin p60 expressed and purified from bacteria (Figure S1). This enzyme was able to sever taxol‐stabilized, control microtubules (with their CTTs intact) but not subtilisin‐treated, taxol‐stabilized microtubules that have the CTT cleaved (‐CTT microtubules, Figure 1a) (Saoudi, Paintrand, Multigner, & Job, 1995). We found that ‐CTT microtubules displayed no severing events, whereas control microtubules displayed 94 events for comparable experimental times.

Despite a lack of severing in experiments using ‐CTT microtubules, we noticed a significant loss of total microtubule polymer (Figure 1). All microtubules in this study were treated with the microtubule‐stabilizing drug taxol, and were otherwise treated exactly the same. Taxol stabilizes microtubules and inhibits microtubule dynamic instability. In the absence of katanin, both control and ‐CTT microtubules lost little polymer (Figure 1a), implying that that taxol was capable of stabilizing the microtubules in our experiments.

To check that ‐CTT microtubules had their CTTs removed, we used SDS‐Page and Western blot analysis (Figure S2). We observed that within the resolution of Coomassie staining, all the tubulin CTTs appeared to be cleaved. We also analyzed the protein content using quantitative Western blotting and found that, within the detectable limits of the method, 100% of the CTTs were removed (Figure S2). Given the resolution of the antibody staining from the linear standard, the data suggest that there were fewer than one CTT for every 1 μm of microtubule length. Thus, the loss of polymer occurred despite a lack of CTTs.

We quantified the loss of polymer using the same metric as prior studies (Bailey et al., 2015; Johjima et al., 2015; Loughlin, Wilbur, McNally, Nédélec, & Heald, 2011) in order to compare our results. In this method, the intensity of the microtubule was measured over time, normalized to the maximum intensity, divided by the background intensity to give a normalized signal‐to‐noise ratio, and plotted over time to find the fraction of polymer as a function time, *F(t)*. The data were fit to an exponential decay of the form:

(1) $$F\left(t\right)=F_{\text{loss}}\exp\left(-\mathrm{kt}\right)+\left(1-F_{\text{loss}}\right)$$

where the fraction of polymer remaining decayed with a characteristic decay rate, *k. F*_loss_ is the maximum fraction of microtubule signal lost, and (1−*F*_loss_) represented the final fraction of microtubule mass remaining in the limit of infinite time (Figure 1b). The decay rate was exponential because the microtubule substrate was being destroyed in the process and thus becomes limiting.

Using fit Equation (1), we first compared the fraction of mass lost, *F*_loss_ for each experiment. As expected, control microtubules without katanin lost only a small fraction of their mass 26 ± 1% (Figure 1b,c, Table S1). This represented the background level of polymer loss of tubulin dimers due to dilution alone for control microtubules—even with the microtubule‐stabilizing agent, taxol, present.

Microtubules lacking the CTT were more stable than that of control microtubules, appearing to gain mass by 7.1 ± 0.2% (fit uncertainty, Figure 1b,c, Table S2). This result was expected, as ‐CTT microtubules have been previously shown to be more stable than control microtubules (Saoudi et al., 1995; Serrano, Avila, & Maccioni, 1984). The increase in 7% was likely due to measurement fluctuations. As it is unlikely for the filament to physically gain mass during the experiment, this number represented the inherent measurement inaccuracy of the imaging system including noise and laser stability. To estimate the accuracy of our measurement, we reported both the uncertainty of the fit parameters to Equation (1) and used 7% as the measurement inaccuracy (Figure 1c, Tables S1 and S2).

We found that the addition of katanin caused control microtubules to lose all of their polymer mass, 103 ± 1% (uncertainty of the fit, Figure 1b,c, Table S1), as expected. Surprisingly, the ‐CTT microtubules lost 55.1 ± 0.8% (uncertainty of the fit, Figure 1b,c, Table S2) when katanin was present despite the fact that subtilisin microtubules could not be severed by katanin. This loss was significantly higher than that of ‐CTT or control microtubules without katanin. The data imply that katanin could cause the loss of tubulin from the polymer, yet at a reduced level compared to control microtubules.

Next, we compared the rate of polymer loss. Biochemical reactions can saturate or run out of reagent during experiments, causing exponential decays in signal, and our experiment was no different (Figure 1b). Instead of using the exponential decay to determine the rate of polymer loss, we used a linear approximation of an exponential decay fit to the first 100 s, when the substrate was not rate limiting:

(2) $$F\left(t\right)=1-\mathrm{kt},$$

to estimate the rate of polymer loss, *k*, for each condition (Figure 1d). The rates of polymer loss (fraction of polymer mass per second) depended on the presence of katanin (Figure 1e). A similar analysis method was employed by Johjima and colleagues to compare enzymatic rates of polymer loss caused by severing enzymes (Johjima et al., 2015).

For control microtubules in the absence of katanin, k = 0.055% ± 0.005% per second (fit uncertainty, Figure 1d,e, Table S3) due to the need to re‐establish a critical monomer concentration in the background after dilution via flow. A similar slow loss was observed for measurements with ADP or mutant katanin previously (Johjima et al., 2015). Even taxol‐stabilized filaments have a critical concentration, albeit low, and dilution resulted in a loss of polymer.

For ‐CTT microtubules in the absence of katanin, we measured an increase in polymer mass at a rate of 0.043% ± 0.006% per second (fit uncertainty, Figure 1d,e, Table S4). As described earlier, it is unlikely for the polymer to grow in diluted conditions, so this rate likely represents a measure of the minimum accuracy of our measurement, assuming ‐CTT microtubules are completely stable in these conditions. In light of this measurement inaccuracy of ±0.04% per second, the rate of loss of control microtubules (0.05% per second) is less noteworthy.

The presence of katanin increased the rate of polymer loss for both control and ‐CTT microtubules. Control microtubules lost polymer at a rate of 0.58% ± 0.01% per second (fit uncertainty, Figure 1d,e, Table S3). The ‐CTT microtubule lost polymer at a rate of 0.48% ± 0.02% per second (fit uncertainty, Figure 1d,e, Table S4). These rates were not identical, yet surprisingly close considering that one set of microtubules was capable of being severed and the other was not.

Taken together, our results indicated that the tubulin CTT was not required for katanin to remove polymer from microtubules. As we did not observe severing of ‐CTT microtubules, our results also indicated that the mechanism of polymer loss was not due to severing of small, diffraction‐limited pieces from near the microtubule ends, as previously proposed (Zhang et al., 2011).

### 2.2 Microtubules lacking CTT were depolymerized by Katanin

Upon inspection, it was clear that the ‐CTT filaments were not being severed but were losing mass from their ends—depolymerization. The loss of polymer metric previously employed did not distinguish between different means of polymer loss.

**Figure 2:**
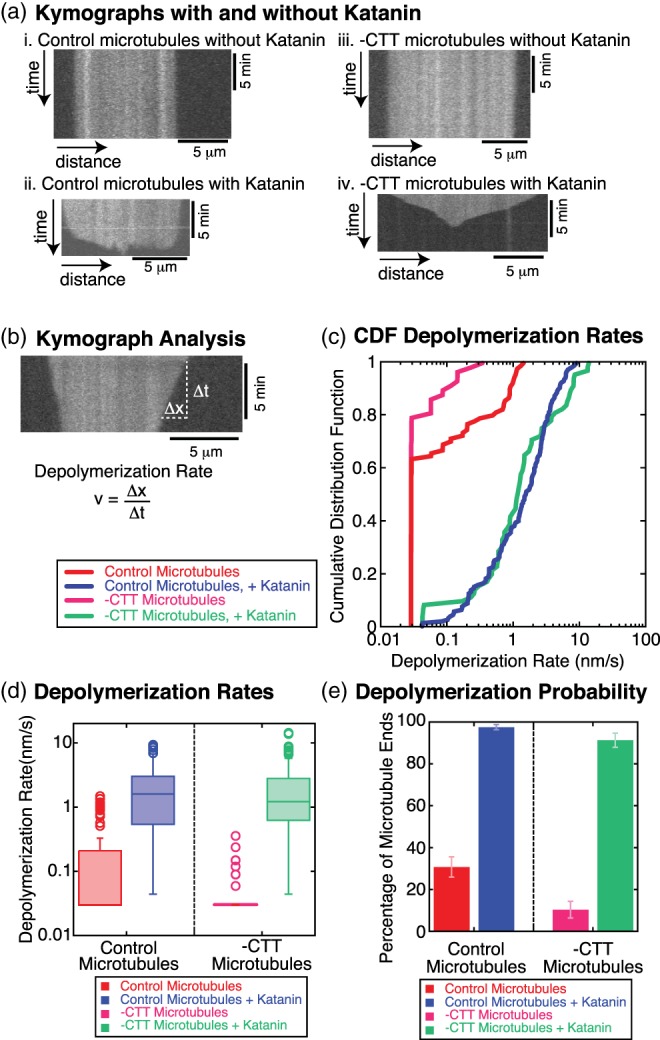
Filament depolymerization depends on the presence of katanin (250 nM). (a) Example kymographs (i) for control microtubules without katanin, (ii) for control microtubules with katanin, (iii) for ‐CTT microtubules without katanin, and (iv) for ‐CTT microtubules with katanin. All microtubules were taxol stabilized. Vertical scale bars 5 min. Horizontal scale bars 5 μm. (b) Example depolymerization rate measurement method using kymograph. The linear loss of polymer from the microtubule end is measured in the x‐direction displacement, Δ*x*. The amount of time it takes to lose the polymer is measured in the y‐direction displacement, Δt. The depolymerization rate is given by v = Δ*x*/Δ*t*. Vertical scale bar 5 min. Horizontal scale bar 5 μm. **(**c) Cumulative distribution plots of the depolymerization rates for control microtubules without katanin (red line, *N* = 91 microtubule ends, 43 microtubules, six chambers), control microtubules with katanin (blue line, N = 161 microtubule ends, 32 microtubules, seven chambers), ‐CTT microtubules without katanin (magenta line, *N* = 58 microtubule ends, 33 microtubules, four chambers), and ‐CTT microtubules with katanin (green line, *N* = 69 microtubules ends, 43 microtubules, six chambers). (d) Box‐whisker plots for control microtubules without katanin (red box), control microtubules with katanin (blue box), ‐CTT microtubules without katanin (magenta box), and ‐CTT microtubules with katanin (green box). On box plots, middle lines of the boxes represent the median and the top and the bottom represent the third and first quartiles, respectively. Open circles represent outlier data. (e)The percentage of filaments that displayed a non‐zero depolymerization rate for control microtubules without katanin (red bar), control microtubules with katanin (blue bar), ‐CTT microtubules without katanin (magenta bar), and ‐CTT microtubules with katanin (green bar). Error bars represent the standard error of proportion. CTT, C‐terminal tail [Color figure can be viewed at wileyonlinelibrary.com]

In order to quantify depolymerization from the ends of microtubules, we used kymographs (space–time projections) to directly measure the change in length as a function of time (Figure 2a,b). The loss of polymer was observed as regions where the white signal from the fluorescent microtubule was removed. For each filament end, we measured the change in the length (horizontal displacement of the signal, Δx) and the time of the change (vertical displacement of the signal, Δt). We divided Δx by Δt to quantify an average depolymerization rate in nanometers per second (Figure 2b).

Microtubules in the absence of katanin showed a low rate of depolymerization in kymographs (Figure 2ai,iii). For control microtubules in the presence of katanin, there was some loss of polymer from the ends, but most of the polymer was lost due to severing, as demonstrated by the jagged bottom edge of the white signal in the kymograph (Figure 2aii). The length of a single pixel in space is 67.5 or 108 nm for our experiments, but the diffraction limit of light for our microscope is approximately 300 nm for the wavelengths we are observing. Any loss of polymer less than four pixels was below the limit of resolution and virtually undetectable. We defined rates to be equivalent to zero if the length of polymer lost was less than four pixels. The measured depolymerization rates that corresponded to “zero” could vary depending on the time of the measurement (see Methods section 4.7 for details).

The depolymerization rate distributions were compared directly using the cumulative distribution plots (Figure 2c). Distributions for the control and ‐CTT microtubules, both taxol‐stabilized, with 250 nM katanin, were indistinguishable using the Kolmogorov–Smirnov statistical test (K‐S test, *p* = 0.1). We plotted the data as box‐whisker plots to further demonstrate that the distributions were similar (Figure 2d). Additionally, we calculated the average depolymerization rates for the control microtubules with katanin, 2.2 nm/s (*N* = 161) and without katanin, 0.24 nm/s (*N* = 91), and for ‐CTT microtubules with katanin, 2.7 nm/s (*N* = 69) and without katanin, 0.051 nm/s (*N* = 58). For this concentration of katanin (250 nM), the rates of depolymerization for microtubules with and without the CTT were equivalent.

In the absence of katanin, most of the filaments did not lose length larger than the resolution of our imaging system, causing a high number of filaments with depolymerization rates effectively equivalent to zero (Figure 2c,d). It was clear that the depolymerization rate distributions were significantly different for control microtubules with katanin compared to without katanin (K‐S test, *p* < 0.001). The depolymerization rate distributions for ‐CTT microtubules with and without katanin were also significantly different (K‐S test, *p* < 0.001; Figure 2c,d). There was no statistical difference between the control microtubules and ‐CTT microtubules both without katanin (K‐S test, *p* = 0.4). These data demonstrated that katanin was necessary to cause microtubule depolymerization.

As a second comparison, we quantified the percentage of filaments that depolymerized for each experimental condition, as defined earlier (Figure 2e). Only 31 ± 6% of control microtubule ends and 10 ± 6% of ‐CTT microtubule ends depolymerized in the absence of katanin. In comparison, nearly all control (96 ± 2%) and ‐CTT microtubules (91 ± 5%) depolymerized in the presence of katanin (Figure 2e). These values were statistically indistinguishable and suggested katanin can depolymerize microtubules lacking the CTT as well as control microtubules at this concentration.

Our results suggest that katanin did not require the CTT to depolymerize microtubules, which is more evidence against the mechanism that depolymerization is caused by the severing of small microtubule pieces from the end.

### 2.3 Depolymerization of microtubules occurred without ATP hydrolysis

**Figure 3:**
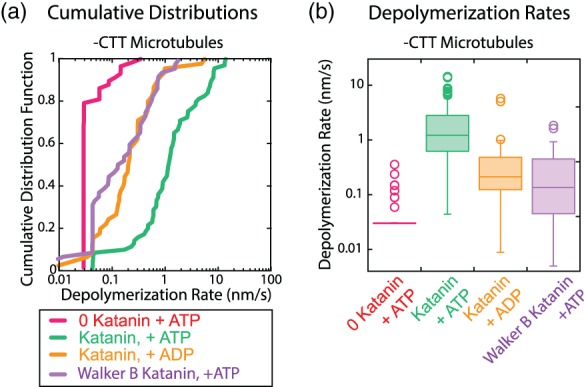
Depolymerization of taxol‐stabilized, ‐CTT microtubules using katanin lacking enzymatic activity. (a) Depolymerization rates plotted as cumulative distributions for ‐CTT microtubules without katanin and with ATP (magenta lines, *N* = 58 microtubule ends, 33 microtubules, four chambers), with katanin and ATP (green lines, *N* = 69 microtubule ends, 43 microtubules, six chambers), with katanin and ADP (orange line, *N* = 45 microtubule ends, 21 microtubules, two chambers), and with Walker B E306Q mutant katanin with ATP (purple line, *N* = 53 microtubule ends, 13 microtubules, three chambers). (b) Box‐whisker plots of the depolymerization rates for ‐CTT microtubules without katanin (magenta box), with wildtype katanin and ATP (green box), with wildtype katanin with ADP (orange box), and with Walker B mutant katanin and ATP (purple box). On box plots, middle lines of the boxes represent the median and the top and the bottom represent the third and first quartiles, respectively. Open circles represent outlier data. All data taken at 250 nM katanin on taxol‐stabilized ‐CTT microtubules. CTT, C‐terminal tail [Color figure can be viewed at wileyonlinelibrary.com]

We previously showed that depolymerization activity of taxol‐stabilized control microtubules catalyzed by katanin required ATP (Díaz‐Valencia et al., 2011; Zhang et al., 2011). Here, we repeated similar experiments quantifying the depolymerization rate distributions for microtubules without the CTTs in the presence of ADP or using a Walker B mutant katanin with ATP (all microtubules were stabilized with taxol). We compared the data to depolymerization rate distributions for ‐CTT microtubules without katanin or with katanin and ATP as negative and positive controls, respectively (Figure 3).

For the ADP data, statistical tests showed that the depolymerization rates of ‐CTT microtubules with ADP (mean speed ADP: 0.51 nm/s) were significantly higher than that of depolymerization speeds without katanin present (mean speed negative control: 0.005 nm/s; *p* < .0001, K‐S test, Figure 3). Depolymerization rates of ‐CTT microtubules with katanin in the presence of ATP were elevated (mean speed positive control: 2.7 nm/s) and statistically different from the ADP condition (*p* < .0001, K‐S test), implying that the process does not require ATP, but it is enhanced by ATP.

We also tested a Walker B mutation (E306Q) in katanin that is capable of binding ATP but not hydrolyzing it. We compared the depolymerization rates of ‐CTT microtubules in the presence of wild type and mutated katanin using cumulative distributions (Figure 3). As we observed with ADP, the Walker B katanin catalyzed the depolymerization of ‐CTT microtubules faster than without katanin (mean speed WalkerB: 0.33 nm/s; *p* < .0001, K‐S test, Figure 3). These rates were still not as high as the rates with wildtype katanin and ATP (*p* < .0001, K‐S test).

Taken together, our results support a mechanism where katanin‐induced depolymerization activity does not require the tubulin CTT, depends on the presence of katanin, and does not require ATP hydrolysis to proceed. We did observe that the presence of ATP enhanced the depolymerization rate of ‐CTT microtubules. These results are interesting because prior results have observed that ATP binding alters the affinity of katanin for the microtubule (Hartman & Vale, 1999) and affects the ability of the AAA+ ring to oligomerize (Hartman et al., 1998). It is possible that the oligomerization state of katanin, as well as the ability to hydrolyze ATP, could enhance the ability of katanin to depolymerize microtubules lacking the CTT.

### 2.4 Depolymerization of microtubules lacking the CTT depended on Katanin concentration

**Figure 4:**
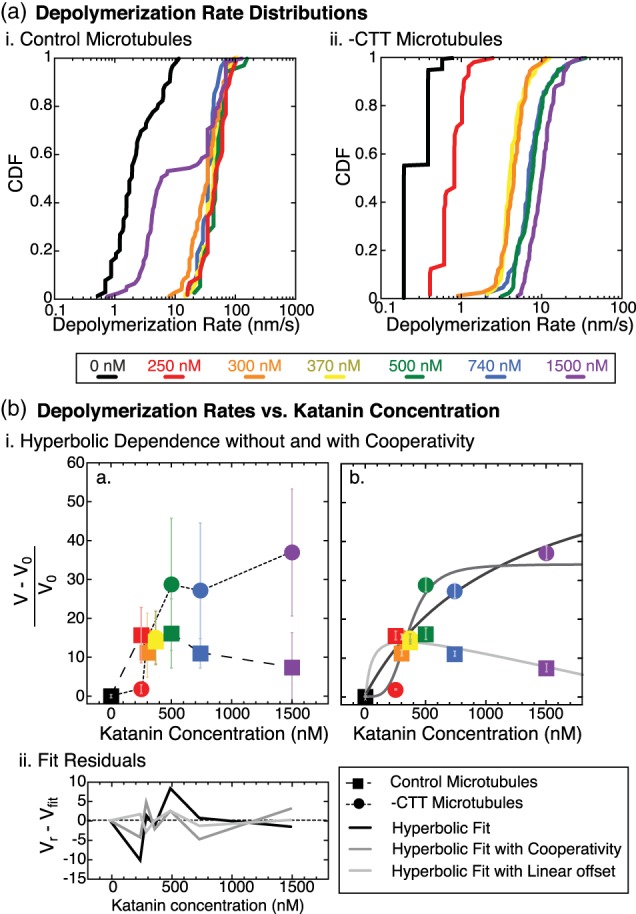
Depolymerization speed as a function of katanin concentration. (a) Cumulative distributions of depolymerization speeds (i) for control microtubules without katanin (black lines, *N* = 63 filament ends) or with katanin at 250 nM (red lines, *N* = 40 ends), 300 nM (orange lines, *N* = 72 ends), 370 nM (yellow lines, *N* = 113 ends), 500 nM (green lines, *N* = 36 ends), 740 nM (blue lines, *N* = 36 ends) and 1.5 μM (purple lines, *N* = 75 ends), and (ii) for ‐CTT microtubules without katanin (black lines, *N* = 242 filament ends) or with katanin at 250 nM (red lines, *N* = 56 ends), 300 nM (orange lines, *N* = 74 ends), 370 nM (yellow lines, *N* = 47 ends), 500 nM (green lines, *N* = 83 ends), 740 nM (blue lines, *N* = 84 ends) and 1.5 μM (purple lines, *N* = 85 ends). All microtubules were taxol stabilized and a single preparation of katanin was used for this data. (b) The relative average depolymerization rate as a function of katanin concentration. (i) (a) All data rescaled by the depolymerization rate in the absence of katanin, V_0_, plotted for ‐CTT microtubules (filled circles) and control microtubules (filled squares). Colors match the colors from part (a). Error bars represent the rescaled standard deviation (unscaled data reported in sFigure S2) to represent the width of the distribution. Dashed lines serve as a guide to the eye. (b) Rescaled depolymerization rates for ‐CTT microtubules were fit to hyperbolic function (Equation (3), black line) and hyperbolic function with cooperativity (Equation 4, dark gray line). Control microtubule data were fit to a difference between a hyperbolic function and a linear equation (Equation (5), light gray line). Fit parameters are given in Tables 5–7. Error bars represent standard error of the mean (uncertainty weighting for the fit). (ii) residuals or each fit found by taking the difference between the relative depolymerization rate, V_r_, and expected depolymerization rate from each fit, V_fit_, plotted as a function of katanin concentration for the hyperbolic function for ‐CTT microtubule data (black line) and hyperbolic function with cooperativity for ‐CTT microtubule data (dark gray line), and hyperbolic fit minus a linear relation for control microtubule data (light gray line). Dashed line represents zero. CTT, C‐terminal tail [Color figure can be viewed at wileyonlinelibrary.com]

We measured the rate of depolymerization as a function of the katanin concentration for both control microtubules and ‐CTT microtubules. For a single preparation of katanin and microtubules, we performed experiments over a range of katanin concentrations from 0 to 1.5 μM (Figure 4). Using kymographs (Figure 2b), the depolymerization rates were quantified for all observable microtubule ends. The distributions of depolymerization rates for each experimental condition were directly compared as cumulative distributions (Figure 4a). It is clear from the cumulative distributions, that the depolymerization rates increase as a function of the katanin concentration.

In order to compare a single number to represent the depolymerization rate distributions, we computed the mean of each distribution and plotted them as a function of the katanin concentration (Figure 4b). We rescaled the depolymerization rate (*V*) by the basal rate (*V*_0_) to find the relative depolymerization rate (*V*_r_):

(3) $$V_{r}=\frac{V-V_{0}}{V_{0}},$$

For the ‐CTT microtubules, the rate of depolymerization depended on the katanin concentration as a hyperbolic that rose monotonically and appeared to saturate (Figure 4b). We fit the relative depolymerization rate for the ‐CTT microtubules to a hyperbolic equation of the form:

(4) $$V_{r}=V_{\max}\frac{1}{1+\left(\frac{K}{\left[\mathrm{kat}\right]}\right)},$$

where *V*_max_ is the maximum relative rate of depolymerization, [kat] is the concentration of the katanin, and *K* is the characteristic rate constant with units of concentration (Figure 4Bi). When we fit the data to Equation 4, we found the *V*_max_ = 70 ± 30 nm/s and *K* = 1,200 ± 900 nM (χ^2^ ~ 180, Figure 4bi, Table S5). The fit was relatively good from the chi‐squared, and the residuals were evenly distributed (Figure 4bii).

As katanin is a hexameric enzyme, it would be reasonable for small oligomers or even full hexamers to be required to cause depolymerization. This oligomerization could manifest as a cooperativity in the depolymerization rates as a function of katanin concentration. To test for signatures of cooperativity, we also fit the data to a hyperbolic function with a cooperativity exponent, of the form:

(5) $$V_{r}=V_{\max}\frac{1}{1+{\left(\frac{K}{\left[\mathrm{kat}\right]}\right)}^{n}},$$

where *n* is the cooperativity exponent (Figure 4bi). When we used Equation (5) to fit to the depolymerization rate data for ‐CTT microtubules, we found the best fit parameters were *V*_max_ = 34 ± 4 nm/s, *K* = 370 ± 40 nM, and *n* = 4 ± 2 (χ^2^ ~ 84, Figure 4b, Table S6). The fit with the cooperativity exponent was better than that of the hyperbolic fit (Equation 4) using the chi‐squared measure. The parameters have lower uncertainties and the residuals are smaller and evenly distributed (Figure 4bii).

The characteristic rate constants that we estimate from our data are likely indicative of the binding constant of katanin for ‐CTT microtubules. We can compare our estimated rate constants to two prior works that measured the *K*_*D*_ value for katanin binding to ‐CTT microtubules. The results of these prior two studies are strikingly different from one another. The first study used microtubule‐pelleting assays and found a low binding affinity of 4 μM (Eckert, Le, Link, Friedmann, & Woehlke, 2012). This K_D_ value is the same order of magnitude as our non‐cooperative model (1.2 μM, Figure 4).

The second study used a wireless‐electrode quartz‐crystal microbalance to measure the binding of katanin to ‐CTT microtubules and they reported much lower *K*_*D*_ value of 6 nM (Johjima et al., 2015). Both of the models to which we fit our data were best fit with reaction rate constants that were higher than those of the second study.

We tried to fit the control microtubule data to Equations 4 and 5, but these equations never fit properly. Instead, we fit the control data to the difference between a hyperbolic function and a linear function of this form:

(6) $$V_{r}=V_{\max}\left(\frac{1}{1+\left(\frac{K}{\left[\mathrm{kat}\right]}\right)}-\frac{\left[\mathrm{kat}\right]}{K^{*}}\right),$$

where *K* ^*^ is a depolymerization rate per katanin concentration that accounted for the linear decrease in the rate. This fit likely implied a competition between two processes that both relied on the katanin concentration. Indeed, active katanin on control microtubules could depolymerize and sever, so the second process could be that of severing, which limits depolymerization. The results recapitulate the same nonmonotonic dependence on katanin as previously measured (Bailey et al., 2015; Díaz‐Valencia et al., 2011).

We found that the best fit values for the rescaled control data had *V*_max_ = 19 ± 8 nm/s, *K* = 51 ± 130 nM, and *K* ^*^ = 2,500 ± 700 nM (χ^2^ ~ 19, Figure 4b, Table S7). The fit appears good according to the low chi‐squared value, but the K fit parameter had a high uncertainty. The residuals were low and evenly distributed (Figure 4bii).

We can compare our control rate constants to binding constants previously measured for control microtubules. The first measured *K*_*D*_ value for katanin binding to control microtubules in the presence of ATPγS was found to be 900 nM and displayed no cooperativity (Hartman & Vale, 1999). In our prior work, we determined an on‐rate (*k*_on_ = 1.6 × 10^6^ M^−1^ s^−1^), binding coefficient (*K*_*D*_ = 45 nM), and a Michaelis–Menten constant (*K*_M_ = 130 nM) for katanin binding to and severing control microtubules (Bailey et al., 2015). The *K* we find for our best fit (51 nM) is quite close to the *K*_*D*_ we previously measured for control microtubules (45 nM) (Bailey et al., 2015).

### 2.5 Katanin binding to microtubules lacking CTTs

**Figure 5:**
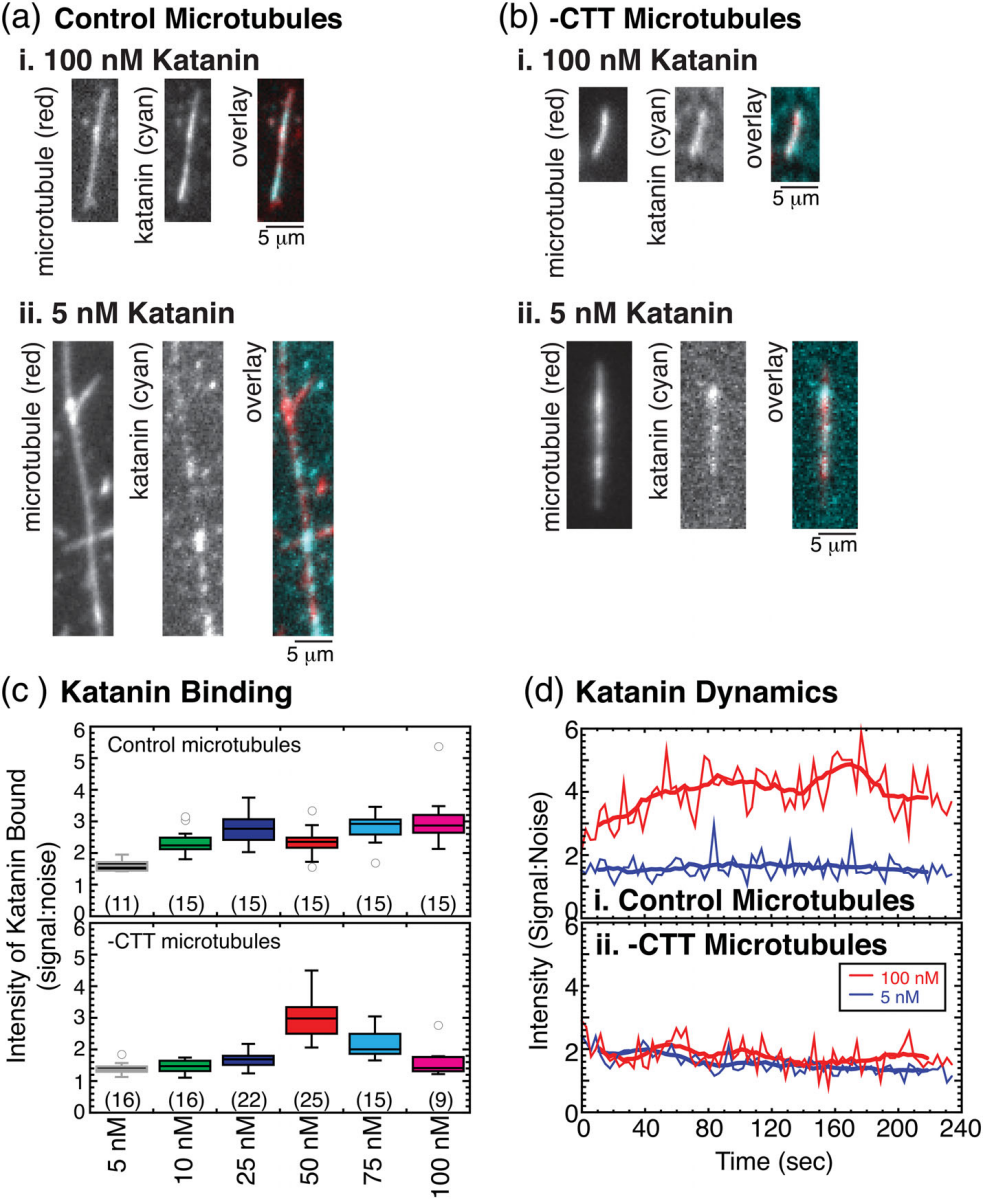
Example of GFP‐katanin binding to microtubules with quantification of the fluorescence intensity. (a) Representative images of control microtubules (left, red) and GFP‐katanin (center, cyan) and color overlay (right) for katanin concentrations (i) 5 nM and (ii) 100 nM. Scale bar 5 μm. All microtubules were taxol stabilized. (b) Representative images of ‐CTT microtubules (left, red) and GFP‐katanin (center, cyan) and color overlay (right) for katanin concentrations (i) 5 nM and (ii) 100 nM. Scale bar 5 μm. All microtubules were taxol stabilized. (c) Quantification of the intensity ratio for katanin binding to microtubules rescaled by the background intensity (signal:noise). Box plots displaying all the intensity data for each measurement of (top) control microtubules and (bottom) ‐CTT microtubules for 5 nM katanin (black box, gray lines), 10 nM katanin (green box, black lines), 25 nM katanin (blue box, black lines), 50 nM katanin (red box, black lines), 75 nM katanin (cyan box, black lines), and 100 nM katanin (magenta box, black lines). Number of measurements taken given in parenthesis in each plot. (d) Quantification of normalized intensity over time of katanin binding to (i) control microtubules or (ii) ‐CTT microtubules. For both plots, data for 5 nM katanin (blue lines) and 100 nM katanin (red lines) are shown. CTT, C‐terminal tail [Color figure can be viewed at wileyonlinelibrary.com]

In order to investigate the mechanism of katanin‐induced depolymerization of ‐CTT microtubules, we purified a green‐fluorescent protein‐labeled katanin and used total internal reflection fluorescence microscopy to directly observe the interaction (Figure 5a,b). The katanin construct we used is a human version of p60 enzyme with a green fluorescent protein (GFP) label on the amino‐terminus. We have previously reported that the rates of katanin‐stimulated microtubule severing are similar for the *Xenopus* and the human versions we used above (Bailey et al., 2015). We found that the human version depolymerizes but does not sever ‐CTT microtubules, as we showed for the *X. laevis* katanin (Figures 1, 2, 3).

At relatively high concentrations (100 nM), GFP‐katanin showed continuous binding along the length of control microtubules (Figure 5ai). At low concentrations (5 nM), katanin formed puncta on the microtubule surface that diffused along the surface, but the concentration was too low to cause severing (Figure 5aii, Figure S4). For microtubules lacking the CTT, the binding of GFP‐katanin at high concentrations (100 nM) coated the filaments (Figure 5bi). For low concentrations of katanin (5 nM), we again observed individual puncta of katanin bound to ‐CTT microtubules (Figure 5bii, Figure S4).

We quantified the intensity bound to the microtubules normalized to the background intensity of katanin in a nearby region (signal:noise, Figure 5c). For each katanin concentration tested, the intensity of binding to control microtubules was significantly different from the intensity on ‐CTT microtubules (Table S8). As is evident from the box plots, the intensities were normally distributed. Data that are normally distributed can be compared using student's *t*‐tests, which confirm the significance results of the K‐S tests (Table S8). Apart from one concentration (50 nM), the control microtubules bound more katanin than ‐CTT microtubules. This implied that the equilibrium dissociation constant, K_D_, was higher for katanin binding to ‐CTT microtubules compared to control, which agreed with prior measurements using bulk binding assays (Eckert et al., 2012).

In addition to quantifying the binding of katanin to microtubules, we imaged the dynamics of katanin binding using single molecule TIRF microscopy (Figure 5d, Figure S4). For high concentrations of katanin (100 nM), the intensity of katanin binding to control microtubules increased and stayed consistently high over a 5‐min movie. The fluorescence of the bound katanin coated the entire microtubule length depolymerizing from the end at early times and severing at later times, as expected (Figure S4ai). When the same concentration of katanin was added to ‐CTT microtubules, the intensity of katanin binding was bright at the beginning but appeared to fade over time (Figure S4bi). Measuring the intensity compared to the background, the loss of signal was comparable to the intensity loss in the background, implying the fading was due to photobleaching (Figure 5d).

At low katanin concentrations (5 nM), individual puncta of GFP‐katanin appeared to associate and dissociate rapidly when control microtubules were imaged with low time resolution (Δt = 3 s, Figure S4). Further, the intensity of katanin on the control microtubule did not appear to fade or bleach over time. On ‐CTT microtubules, the low katanin concentration again appears to fade over time due to photobleaching (Figure S4). Upon inspection of the high time resolution data (Δt = 0.06 s), it was clear that the individual katanin puncta on the control microtubules are highly mobile, diffusing along the filament, and were observed to associate and dissociate (Figure S4aii), which has been previously demonstrated (Eckert et al., 2012). For the ‐CTT microtubules, the individual puncta were not mobile (Figure S4bii) and appeared to photobleach over time.

The imaging conditions were identical for all data sets measuring GFP‐katanin dynamics, implying that the photobleaching effects should have been the same during imaging. The observation that GFP‐katanin on control microtubules did not appear to bleach suggested that a dynamic equilibrium was established with the katanin in solution. With a supply of new katanin, the overall intensity stayed constant or even increased over time compared to the background noise.

The intensity was not constant for the katanin bound to ‐CTT microtubules. Although ‐CTT microtubules bound fewer katanin molecules, they stayed bound to the microtubule and turned‐over slower with katanin in solution. Thus, the katanin on ‐CTT microtubules was bound long enough to photobleach. These data implied that the off rate for katanin was higher for control microtubules than for ‐CTT microtubules. Assuming that the on‐rate is diffusion limited for both control and ‐CTT microtubules at equilibrium, these data could be interpreted to suggest that the GFP‐katanin *K*_*D*_ for control microtubules should be higher than that of the *K*_*D*_ for ‐CTT microtubules. Indeed, this conclusion was reached by a prior study using wireless‐electrode quartz‐crystal microbalance (Johjima et al., 2015).

Our imaging data could help to clear up the controversy that two different groups measured opposite effects on the binding affinity of katanin for control and ‐CTT microtubules (Eckert et al., 2012; Johjima et al., 2015). Specifically, bulk binding showed katanin K_D_ increased for ‐CTT microtubules compared to control (Eckert et al., 2012), while measurements with a microbalance showed the K_D_ decreased for ‐CTT compared to control (Johjima et al., 2015). If the two different methods measure different aspects of binding, specifically the total binding mass or the equilibrium off‐rate, they could result in opposite trends, as reported. Our direct visualization experiments can observe both of these effects: the increased binding to control microtubules and the reduced off‐rate to ‐CTT microtubules, supplying a rationale for the incompatible results of the prior studies.

## 3 DISCUSSION

**Figure 6:**
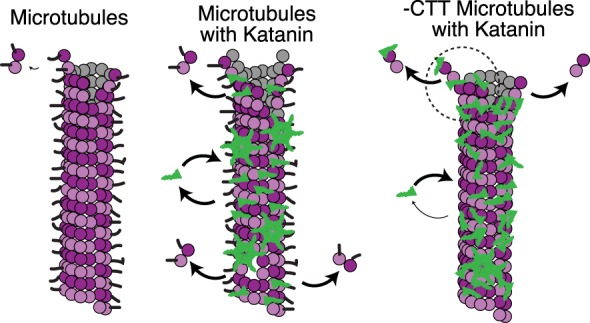
Cartoon mechanism of microtubule depolymerization of ‐CTT microtubules. (left) Control microtubules (magenta) in the absence of katanin lose dimers from the ends due to normal degradation to replenish the background concentration. This loss of polymer is slow. (middle) In the presence of katanin (green) katanin can catalyze the loss of dimers from both the ends of control microtubules, called depolymerization, and from the middle of control microtubules, called severing. We observe a significant amount of mobility, association, and dissociation of katanin to and from the filaments. (right) Microtubules lacking the CTT can still bind katanin, but fewer katanins are bound and the bound katanin is less mobile. Without the CTT, katanin cannot sever microtubules but can still catalyze the loss of dimers from the ends. CTT, C‐terminal tail [Color figure can be viewed at wileyonlinelibrary.com]

Our data support a model where katanin's abilities to sever and depolymerize microtubules occur through separate mechanisms. Specifically, our data suggest that depolymerization is not equivalent to the severing of small regions of microtubule from the end, as previously suggested (Zhang et al., 2011). This conclusion is supported by several novel results we present here (a) microtubules lacking the CTT cannot be severed but still lose polymer by a process of katanin‐catalyzed depolymerization (Figures 1 and 2), (b) depolymerization does not require ATP hydrolysis, and depolymerization rates are enhanced in the presence of ATP (Figure 3), (c) the depolymerization rate correlates to katanin concentration (Figure 4), and (d) katanin is directly observed to bind to microtubules during depolymerization (Figures 5 and 6).

Interestingly, both the data on the depolymerization rate as a function of katanin (Figure 4) and the binding intensity measurements (Figure 5) indicated that katanin can likely bind to microtubules by two means. For control microtubules, the binding affinity was tighter (51 nM) compared to ‐CTT microtubules (370 nM to 1.2 μM), which agreed with one prior study that also measured the *K*_*D*_ of katanin to ‐CTT microtubules (Eckert et al., 2012). The direct imaging of GFP‐katanin revealed that more katanin can bind to control microtubules, and katanin diffused on the surface of control microtubules, as previously reported (Eckert et al., 2012). Despite more binding, katanin was more dynamically associated with control microtubules, revealed by the fact that the bound katanin does not photobleach as quickly on control microtubules, implying the katanin could turnover with free katanin in solution.

Katanin binding to ‐CTT microtubules had a lower concentration bound, corresponding to the higher K_D_ value, but bound katanin was static and unable to diffuse. The static katanin also photobleached because it was not exchanging with the soluble pool in solution. These results agreed with a prior study that reported tight binding of katanin to ‐CTT microtubules (Johjima et al., 2015). Our new imaging data could help to uncover the reason for the previous discrepancy in the literature (Eckert et al., 2012; Johjima et al., 2015) and pointed to katanin having more than one binding mode or binding site to tubulin in microtubules.

There was prior evidence for multiple modes of katanin binding to microtubules from structural work, which suggested that katanin had a second microtubule‐binding site in addition to the tubulin CTT (Zehr et al., 2017). Their proposed mechanism was that multivalent binding occurs between katanin and the microtubule. The katanin hexamer, through ATP binding and hydrolysis, can bend and straighten to enable wedging of the dimers from the side of the filament (Zehr et al., 2017). Such a scheme could only work if other parts of the katanin, away from the CTT‐binding pore loops, were also able to bind to the microtubule surface.

In addition to these structural studies, molecular dynamics simulations have suggested that it would be easier to remove a dimer by pushing into the lattice, rather than pulling to unravel a dimer (Barsegov et al., 2017; Jiang, Bailey, et al., 2017b; Theisen, Desai, Volski, & Dima, 2013). Such computational results are additional evidence that the unfoldase mechanism is less likely than a wedging or pushing mechanism. Our new data are consistent with a wedging mechanism, considering that end dimers can be extracted without the presence of the CTT.

Given the evidence presented in prior works, and our new results here, we propose that katanin could remove tubulin dimers from microtubules through a mode that uses wedging between the dimers to dislodge them from the microtubule tips but not from the body (Figure 6). From our imaging data, this alternative binding site cannot accommodate the binding of as many katanin molecules as the CTT interaction, yet the binding appears static (Figure 5). It is possible that the second binding site has a lower stoichiometry than that of the CTT binding site. The removal of end dimers we observe is also passive, because dimers were removed in the absence of ATP. This proposed mode of dimer removal would be helpful to dissociate dimers during regular severing as well, which requires both the ATPase activity and the CTT.

The mechanism we propose for passive katanin wedging causing depolymerization is similar to that proposed for actin severing by ADF‐cofilin, which does not use ATP. ADF‐cofilin severs actin by binding to the side of the actin filament, loosening the bonds between neighboring monomers, and ultimately cutting the filament (McCullough et al., 2011; McCullough, Blanchoin, Martiel, & De la Cruz, 2008). Katanin‐induced depolymerization may work in a similar manner—simply binding and wedging between the dimers until they pop off the end. This mechanism works because the barrier to remove a single dimer from the filament end is lower than from the body due to the lack of neighboring dimers. Further, the fluctuations of the dimers at the ends should be significantly higher compared to dimers within the lattice. Fluctuations could allow katanin to bind to the interdimer interfaces to remove the dimer.

Microtubule severing and depolymerization are important and highly regulated processes in cells. Our work highlights that these two processes can both be catalyzed by katanin but likely occur via different mechanisms because depolymerization can occur on microtubules lacking the CTT, but severing cannot. Both severing and depolymerization activities may take advantage of a proposed second binding site and wedge mechanism to remove dimers. Only depolymerization may work through passive means not requiring ATP hydrolysis. Future experiments could test this model using modified proteins with altered binding affinities for the secondary tubulin binding site or microtubules that are held together tighter through different stabilization methods.

## 4 EXPERIMENTAL PROCEDURES

### 4.1 Protein purification

The enzymatic katanin p60 construct was a gift from the Heald lab (20). The construct is a full‐length *Xenopus laevis* katanin p60 with a maltose binding protein (MBP) as the affinity tag on the N‐terminus. For data comparing depolymerization rates as a function of the concentration of katanin and quantifying katanin binding, we used a human p60 isoform with a green fluorescent protein (GFP) tag and maltose binding protein (MBP) tag for purification. We have previously shown that these two katanin constructs have similar high severing activity (Bailey et al., 2015). We transformed the plasmid into BL21 competent *Escherichia coli* (New England BioLabs, Waltham, MA), grew a 5 mL liquid culture for 3–4 hr, and added it to a 400 mL culture in the afternoon. The large culture grew at 37 °C until it reached an OD of 0.8. We induced protein production with 1 mM IPTG and continued to grow the culture at 16 °C for 16 hr. Cells were pelleted and lysed in resuspension buffer (20 mM Hepes pH 7.7, 250 mM NaCl, 0.5 mM BME, 10% glycerol, 0.25 mM ATP) via sonication. The lysate was incubated at 4 °C with amylose resin (New England BioLabs, Waltham, MA) for 1.5 hr. We poured the lysate/resin mixture into a gravity column and allowed excess lysate to pass through the column. The column was washed 15 mL of resuspension buffer (20 mM Hepes pH 7.7, 250 mM NaCl, 0.5 mM BME, 10% glycerol, 0.25 mM ATP). The protein was eluted in 5 mL elution buffer (20 mM Hepes pH 7.7, 250 mM NaCl, 0.5 mM BME, 10% glycerol, 0.25 mM ATP, 10 mM maltose). We obtained protein concentration using a Bradford assay. We ran an SDS‐Page gel and stained with Coomassie blue to check protein purity and found it was 98% pure (Figure S1).

### 4.2 Taxol‐stabilized microtubules

Control, taxol‐stabilized microtubules were made by combining a 1:3–1:20 ratio of labeled rhodamine tubulin (Cytoskeleton Inc., Denver, CO) or Dylight 649 (Thermo Fisher Scientific, Waltham, MA) tubulin with purified unlabeled tubulin made from porcine brains in house using the method described in (Peloquin et al., 2005). We resuspended both the unlabeled and labeled tubulin in PEM‐100 (100 mM K‐Pipes, pH 6.8, 2 mM MgSO_4_, 2 mM EGTA) to a concentration of 5 mg/mL (45.5 μM) and incubated it on ice for 10 min. We combined the labeled and unlabeled tubulin and centrifuged at 366,000*g* at 4 °C for 10 min to remove aggregated or insoluble tubulin. We added 1 mM GTP to the tubulin and incubated at 37 °C for 40 min to polymerize the microtubules. We added 50 μM taxol and incubated for 40 min at 37 °C to stabilize the microtubules. We centrifuged the microtubules at 16,200*g* at 27 °C for 10 min to clarify the microtubules. We resuspended the microtubule pellet in PEM‐100 and 50 μM taxol.

### 4.3 Subtilisin‐treated taxol‐stabilized microtubules

We created microtubules lacking the carboxy terminal tails of both alpha and beta tubulin by treating microtubules with subtilisin. After we polymerized taxol microtubules, we incubated them with 100 μg/mL subtilisin for 45 min. We stopped the reaction using 2 mM PMSF. We further removed the subtilisin by centrifuging the microtubules for 30 min at 16,200*g* 27 °C. We resuspended the pellet in PEM‐100 and 50 μM taxol.

### 4.4 In vitro assays

We used in vitro microscopy assays to quantify the loss of polymer and depolymerization rates. Coverslip cleaning and silanization with 2% dimethyldichlorosilane (GE Healthcare, Chicago, IL) are identical to previously published protocols (Diaz‐Valencia, Bailey, & Ross, 2013; Dixit & Ross, 2010). Silanized coverslips are assembled onto slides to make flow chambers using double‐sided tape as the spacer and adhesive. Once assembled, we flowed 10 μL washes to perform our experiment. We incubated an antibody to tubulin, MAB1864 (MilliporeSigma, Burlington, MA) at 2% (w/v) in Katanin Activity Buffer (20 mM Hepes pH 7.7, 10% glycerol, 2 mM MgCl_2_) for 5 min to enable binding of microtubules to the surface. We added 5% (w/v) Pluronic F‐127 in Katanin Activity Buffer (20 mM Hepes pH 7.7, 10% glycerol, 2 mM MgCl_2_) to block the surface. We flowed in a 1:100 dilution of labeled, taxol‐stabilized microtubules and incubated in the chamber for 5 min to allow them to adhere. The concentration of taxol was 20–50 μM. We flowed in an Enzymatic Mix (20 mM Hepes pH 7.7, 10% glycerol, 2 mM MgCl_2_, 2 mM ATP, 0.025 mg/mL BSA, 0.05% F‐127, 20 μM taxol, 10 mM DTT, 15 mg/mL glucose, 0.15 mg/mL catalase, 0.05 mg/mL glucose oxidase) to flow out excess microtubules. Please note that all the microtubules are taxol stabilized throughout the entire experiment, as taxol is present in all buffers when microtubules are present.

### 4.5 Imaging

We captured images using a Nikon Ti‐E microscope which has a ×60 objective (NA 1.49) coupled with an external ×4 expander to a final pixel size of 67.5 nm or a ×2.5 expander for a final pixel size of 108 nm on an iXon EM‐CCD 512x512 camera (Andor Technology, Belfast, United Kingdom). The microscope has an Intenselite XeHg light source for illumination in the epi‐fluorescence path, which allowed us to image microtubules. Imaging of GFP‐katanin binding was performed using a home‐built total internal reflection fluorescence (TIRF) microscope illuminated with a 50 mW 488 nm laser. We imaged the chamber prior to adding katanin to check the microtubules and to make sure our imaging conditions are not damaging the microtubules. We captured images of fluorescent microtubules every 3–5 s with shuttering between. We recorded for 3 min before flowing in the experimental buffer containing either no katanin or 35–1,500 nM katanin (wild‐type katanin) in enzymatic mix supplemented with oxygen scavengers (15 mg/mL glucose, 0.15 mg/mL catalase, and 0.05 mg/mL glucose oxidase). Movies were taken at 3–5 s intervals for 10 min. Imaging of microtubules and GFP‐katanin was performed using alternating illumination between epi‐fluorescence and TIRF.

### 4.6 Loss of polymer data analysis

Loss of polymer analysis was performed in ImageJ as previously described (Bailey et al., 2015). Time series data (movies) were imported into ImageJ as nd2 files (Nikon, Tokyo, Japan) using the LOCI plugin. We used the line tool to draw a segmented line, three pixels wide, over the length of the microtubule. We used the macro “measure stacks” to measure the mean intensity of the line for each frame of the movie. The same line was moved to a location without microtubules to measure the mean background intensity near the microtubules. The intensity was normalized by dividing the intensity by the background (I_measured_/I_BG_) to give the signal to noise ratio. In order to remove the background, we subtracted one (1 = I_BG_/I_BG_). This caused all the initial intensities to be one. The individual, normalized microtubule intensity data were then averaged together and the error bars represent the standard error of the mean over multiple measurements of different filaments. There is some variability in the measured fluorescence, as described in the main text. This is most likely due to the laser power fluctuating over time from experiment to experiment. As well as the fact that the normalization described above is performed for each microtubule separately, introducing some stochastic noise into the final average. The intensity fluctuations have the largest effect on the measurement of the final microtubule polymer fraction remaining found by fitting to Equation (1). Because of this, we have quoted the uncertainty of the fits from the data in the text but plotted the uncertainty in the measurement as 7% in Figure 1c.

The number of microtubules analyzed for each experimental parameter are given in the figure captions. The data were fit to exponential (Equation (1)) or linear (Equation (2)) rate equations using least squares fitting routine in KaleidaGraph. The fit parameters with uncertainty of each fit, and goodness‐of‐fit parameters are given in Tables S1–S4.

### 4.7 Depolymerization speed analysis

Depolymerization speed analysis was performed in FIJI/ImageJ. We used the line tool to draw a three‐pixel wide line over the microtubule and used the “Reslice” function to make a kymograph with distance on the *x* axis and time on the *y* axis. Alternatively, we also used the “Multi‐Kymograph” tool and chose a line width of three pixels. These tools are equivalent. The microtubule polymer loss has a good signal‐to‐noise ratio in kymographs (Figures 2 and 6). The box tool provided us with the number of pixels of the height and width of the box corresponding to the time and distance of depolymerization, respectively. The pixel conversion in the *x* axis is 67.5 nm per pixel or 108 nm per pixel, depending on the image expander used. The pixel conversion in the y axis is 5 s per pixel. We calculated the depolymerization speed in nm/s by dividing the distance by the time. Given the diffraction limit of our system is ~300 nm, any change in distance that was four pixels or less was within the diffraction limit and equivalent to zero change in displacement. The lengths of different movies varied slightly, causing the minimum measurable velocity to differ slightly from movie to movie and experiment to experiment, since *v* = Δ*x*/Δ*t*, and the cut‐off is only determined by the Δ*x*. For example, two data sets, one with Δ*t* = 10 s and one with Δ*t* = 5 min would have different velocities if they both only lost 1 pixel = 67.5 nm of length, i.e., 6.75 nm/s and 0.23 nm/s, respectively. Thus, both of these velocities are below the resolution limit in the *x* direction and are effectively equal to zero because Δ*x* < 4 pixels. Thus, there is no specific velocity cutoff, but there is a spatial threshold for what is classified as “zero” velocity.

Each end was measured separately and averaged. The number of ends measured per microtubule depended on the severing by katanin, which could make two new ends per severing event. The number of individual microtubule ends measured is given in the figure caption for each experiment type.

### 4.8 Quantification of Katanin binding

The imaging of katanin binding was performed using a home‐built total internal reflection fluorescence microscope. Time series movies of katanin binding and activity on microtubules were recorded in both the red (microtubule) and green (GFP‐katanin) channels. After katanin was added, and bound to the filaments, a single frame was used to quantify the intensity of the microtubules and the intensity of katanin. A region of interest along the length of the filament was chosen and used to measure the integrated intensity of katanin. The same region was shifted off the filament to measure the background intensity nearby. The signal intensity of each filament was divided by the noise intensity for the nearby background measurement. The ratio of katanin to microtubule signal‐to‐noise measurements were computed for each filament and plotted in Figure 5c. Using the same data, we can find the normalized intensity of katanin bound to microtubules over time, as reported in Figure 5d. A complete example with kymograph data are shown in supplemental Figure S4c.

## CONFLICTS OF INTEREST

The authors declare that they have no conflicts of interest with the contents of this article.

## AUTHOR CONTRIBUTIONS

M.E.B. designed and performed experiments, analyzed data, and drafted the manuscript. M.A.T. performed experiments, analyzed data, and drafted the manuscript. L.B. performed experiments and analyzed data. A.K. analyzed data. J.L.R. designed experiments, analyzed data, and drafted and edited the manuscript.

## Supporting information


**Supplemental Table 1** Supporting Information

## Data Availability

The data that support the findings of this study are available from the corresponding author upon reasonable request.
