# Supplementary material for: Katanin catalyzes microtubule depolymerization independently of tubulin C‐terminal tails
Source: Cytoskeleton (Hoboken). 2019 May 2;76(3):254–68. doi: 10.1002/cm.21522 (PMC6618852; doi:10.1002/cm.21522)

**Katanin Catalyzes Microtubule Depolymerization Independent of Tubulin Carboxy Terminal Tails**

Liudmila Belonogov^1^, Megan E. Bailey^2^, Madison A. Tyler^3^, Arianna Kazemi^1^, Jennifer L. Ross^1,3^*

^1.^ Department of Physics, University of Massachusetts, Amherst

^2.^ Department of Electrical Engineering and Information Technology TU Darmstadt, Darmstadt, Germany

^3.^ Molecular and Cellular Biology Graduate Program, University of Massachusetts, Amherst

* Corresponding author

**Supplemental Table 1: Fits from Figure 1Bi**

 (Eq. 1)

|  | Fit | Chi Sq | F_loss_ | k (1/s) | % Total LOP |
| --- | --- | --- | --- | --- | --- |
| Control MTs | Eq. 1 | 0.013 | 0.26 ± 0.01 | 0.0021 ± 0.0002 | 26 ± 1 |
| Control MTs + WT katanin | Eq. 1 | 0.160 | 1.03 ± 0.01 | 0.0087 ± 0.0003 | 103 ± 1 |

**Supplemental Table 2: Fits from Figure 1Bii**

 (Eq. 1)

|  | Fit | Chi Sq | F_loss_ | k (1/s) | % Total LOP |
| --- | --- | --- | --- | --- | --- |
| -CTT MTs | Eq. 1 | 0.027 | -0.071 ± 0.002 | 0.009 ± 0.001 | -7.1 ± 0.2 |
| -CTT MTs + WT katanin | Eq. 1 | 0.104 | 0.551 ± 0.008 | 0.0114 ± 0.0006 | 55.1 ± 0.8 |

**Supplemental Table 3: Fits from Figure 1Di**

, (Eq. 2)

|  | Fit | Goodness of Fit: R^2^ | Chi Sq | k (1/s) |
| --- | --- | --- | --- | --- |
| Control MTs | Eq. 2 | 0.710 | 0.0024 | 0.00055 ± 0.00005 |
| Control MTs + WT katanin | Eq. 2 | 0.980 | 0.0138 | 0.0058 ± 0.0001 |

**Supplemental Table 4: Fits from Figure 1Dii**

, (Eq. 2)

|  | Fit | Goodness of Fit: R^2^ | Chi Sq | k (1/s) |
| --- | --- | --- | --- | --- |
| -CTT MTs | Eq. 2 | 0.556 | 0.005 | -0.00043 ± 0.00006 |
| -CTT MTs + WT katanin | Eq. 2 | 0.721 | 0.065 | 0.0048 ± 0.0002 |

**Supplemental Table 5: Hyperbolic fits to raw data and rescaled data for Figure 4Bib and Supplemental Figure 2 fit to -CTT data**

, Eq 3.

|  | Fit | Chi Sq | A (nm/s) | K (nM) |
| --- | --- | --- | --- | --- |
| Raw Data | Eq. 3 | 15.426 | 20 ± 8 | 1100 ± 800 |
| Rescaled Data | Eq. 3 | 179.53 | 70 ± 30 | 1200 ± 900 |

**Supplemental Table 6: Hyperbolic fits with cooperativity to raw data and rescaled data for Figure 4Bib and Supplemental Figure 2 fit to -CTT data**

, Eq 4

|  | Fit | Chi Sq | v_max_ (nm/s) | K (nM) | n |
| --- | --- | --- | --- | --- | --- |
| Raw Data | Eq. 4 | 7.4 | 10 ± 1 | 360 ± 40 | 4 ± 2 |
| Rescaled Data | Eq. 4 | 84.8 | 34 ± 4 | 370 ± 40 | 4 ± 2 |

**Supplemental Table 7: Hyperbolic fits with linear loss term to raw data and rescaled data for Figure 4Bib and Supplemental Figure 2 for control data**

, Eq 5

|  | Fit | Chi Sq | V_max_ (nm/s) | K (nM) | K* (nM) |
| --- | --- | --- | --- | --- | --- |
| Raw Data | Eq. 5 | 202 | 60 ± 30 | 50 ± 120 | 2700 ± 800 |
| Rescaled Data | Eq. 5 | 19.4 | 19 ± 8 | 50 ± 130 | 2500 ± 700 |

**Supplemental Table 8: Data from Figure 5Cii comparing the probabilities that the intensity of katanin binding to control and -CTT microtubules is distinct.**

|  | **Control Microtubules** | | | **-CTT Microtubules** | | | **p-value** | |
| --- | --- | --- | --- | --- | --- | --- | --- | --- |
| **Katanin Concentration (nM)** | **Average Value** | **Standard Deviation** | **N** | **Average Value** | **Standard Deviation** | **N** | **KS Test** | **T-test** |
| **5** | 1.6 | 0.2 | 11 | 1.4 | 0.2 | 16 | 0.010 | 0.007 |
| **10** | 2.3 | 0.4 | 15 | 1.5 | 0.2 | 16 | <0.001 | <0.0001 |
| **25** | 2.8 | 0.5 | 15 | 1.7 | 0.3 | 22 | <0.001 | <0.0001 |
| **50** | 2.4 | 0.5 | 15 | 3.0 | 0.6 | 25 | 0.002 | 0.0005 |
| **75** | 2.8 | 0.4 | 15 | 2.2 | 0.4 | 15 | 0.005 | 0.0006 |
| **100** | 3.0 | 0.8 | 15 | 1.6 | 0.5 | 9 | <0.001 | <0.0001 |

**Supplemental Figure 1. SDS-Page gel of katanin preparations.** Example protein preparation diagnostic SDS-Page gel to show the preparation steps. Gel was composed of 7% polyacrylamide in the bulk and stained with Coomassie blue to reveal all protein. The preparation steps are labeled. For clarity, Lane 1: molecular weight ladder, Lane 2: uninduced bacteria cells, Lane 3: induced bacteria cells, Lane 4: lysed cell supernatant added to beads, Lane 5: flow through of protein that did not stick to beads (we can use this for a second preparation, since much of the protein did not bind), Lane 6: wash of beads, Lane 7: elusion 1, Lane 8: elution 2 (used for experiments), Lane 9: elution 3 (used for experiments), Lane 10: elution 5, Lane 11: elution 4. Elution fractions used were the second and third, which were combined. Concentration was determined by densitometry of second SDS-Page gel by comparing to BSA standards of known concentration or using a Bradford assay.

**Supplemental Figure 2. Western blots of -CTT microtubule preparations.** To determine a quantitative upper bound on the percentage of CTT cleaved by subtilisin treatment, we ran a SDS-Page protein gel and stained for CTT with anti-CTT antibody staining (DM1A, Millipore). **(Top)** Ponceau staining of SDS-Page gel after transfer to methylcellulose membrane. Lane 1: molecular weight marker with stained molecular weights labeled (Bio-Rad). Lane 2: skipped. Lanes 3 – 6: control tubulin with 18 nM, 36 nM, 55 nM, and 73 nM control tubulin. Lane 7: skipped. Lanes: 8 – 12: -CTT microtubules at 38 µM (lane 8), 18 µM (lane 10), and 11 µM (lane 12), with lanes 9, 11 skipped. **(Bottom)** Anti-CTT staining of the same membrane. Only staining for -CTT visible in lanes 3 – 6: control tubulin. Lane 3, 18 nM, is barely visible. None of the subtilisin-treated tubulin is visible by antibody, implying that there is less than 18 nM of the -CTT microtubules with a CTT. We estimate the minimal fraction of CTTs remaining as:

From this fraction, we can estimate the minimal distance, in µm, between remaining CTTs in a microtubules with 13 protofilaments and 8 nm long dimers.

We find that the minimum distance estimated is 1.3 µm between CTT. This is the upper bound on the average distance between CTTs remaining on subtilisin-treated microtubules as determined from our Western blotting. It is possible that the distance between CTTs remaining could be even higher than this estimate. Any remaining CTTs would also be stochastically distributed, so this estimate is only an average over the population.

**Supplemental Figure 3. Depolymerization rates as a function of GFP-Katanin concentration with fits for control and -CTT microtubules plotted in as original and rescaled data. (A) (i)** Original depolymerization rates plotted as a function of katanin concentration for control microtubules (filled squares) and -CTT microtubules (filled circles). Colors correspond to the color scheme of figure 4 in the main text, and the data is the same, so the number of experimental measurements are also identical to that in Figure 4. Dashed lines are a guide to the eye. **(ii)** Same data as in (i) with fits to the -CTT data that are hyperbolic (Eq. 3, black line), or hyperbolic with cooperativity (Eq. 4, dark gray line) and with fit to the control data that is the difference between a hyperbolic function and a linear function (Eg. 5, light gray line). Fit parameters are given in Supplemental Tables 5-7. **(B)** Difference between the depolymerization rate, V, and expected depolymerization rate from each fit, V_fit_, plotted as a function of katanin concentration for the hyperbolic function fit to -CTT microtubule data (black line), hyperbolic function with cooperativity fit to -CTT microtubule data (dark gray line), or hyperbolic function minus a linear function fit to control data (light gray line). Dashed line represents zero.

**Supplemental Figure 4. Examples kymographs with GFP-Katanin binding to microtubule ends.**

**(A)** Control microtubules example kymographs and quantification of intensity over time.

**(i)** Example kymographs of GFP-katanin imaged using TIRF microscopy bound to control microtubules in the presence of 100 nM GFP-katanin (look-up table set to 0 – 9000). For each concentration, two kymographs are represented.

**(a)** Kymograph displays the total 5 minutes of imaging. For 100 nM, the intensity of the GFP-katanin is fairly constant and depolymerization is obvious as well as severing around 4 minutes. The yellow dashed box is the 1-minute interval represented by the kymograph on the right (in ***b***). Horizontal scale bar is 5 µm; vertical scale bar is 1 min.

**(b)** Kymograph made from the same exact microtubule and imaging as the left kymograph, with all frames displayed taken at an exposure time of 0.06 s; the total length of time represented in the vertical direction is 1 min. The time of the right kymograph is the same minute outlines in the yellow dashed box on the left kymograph (in ***a***). The fluctuations in intensity are more obvious in the higher time resolution kymograph. Due to the size of this kymograph, it has been rescaled by 50% compared to the left kymograph. Horizontal scale bar is 5 µm; vertical scale bar is 3 s. The look up tables (LUT) for all kymographs is set to the same level of 0-9000 in a 32-bit image prior to conversion to 8-bit for displaying.

**(c)** Quantification of the intensity of the GFP-katanin on the microtubule over time. The intensity profile, averaged over 10 pixels along the microtubule length was measured over time using the low time resolution kymographs. Regions of interest on the microtubule (green line, microtubule) and off the microtubule (black line, background) were quantified and plotted over time.

**(ii)** Example kymographs of GFP-katanin imaged using TIRF microscopy bound to control microtubules in the presence of 5 nM GFP-katanin (look-up table set to 0 – 800). For each concentration, two kymographs are represented.

**(a)** Kymograph displays the total 5 minutes of imaging. For 100 nM, the intensity of the GFP-katanin is fairly constant and depolymerization is obvious as well as severing around 4 minutes. The yellow dashed box is the 1-minute interval represented by the kymograph on the right (in ***b***). Horizontal scale bar is 5 µm; vertical scale bar is 1 min.

**(b)** Kymograph made from the same exact microtubule and imaging as the left kymograph, with all frames displayed taken at an exposure time of 0.06 s; the total length of time represented in the vertical direction is 1 min. The time of the right kymograph is the same minute outlines in the yellow dashed box on the left kymograph (in ***a***). The fluctuations in intensity are more obvious in the higher time resolution kymograph. Due to the size of this kymograph, it has been rescaled by 50% compared to the left kymograph. Horizontal scale bar is 5 µm; vertical scale bar is 3 s. The look up tables (LUT) for all kymographs is set to the same level of 0-9000 in a 32-bit image prior to conversion to 8-bit for displaying.

**(c)** Quantification of the intensity of the GFP-katanin on the microtubule over time. The intensity profile, averaged over 10 pixels along the microtubule length was measured over time using the low time resolution kymographs. Regions of interest on the microtubule (green line, microtubule) and off the microtubule (black line, background) were quantified and plotted over time.

**(iii)** Quantification of the intensity normalized to the background for GFP-katanin binding along control microtubules over time. Data from parts **(i,c and ii,c)** are divided to give the signal (microtubule) to noise (background) for 100 nM (red line) and 5 nM (blue line). Thick lines represent averaged data over 30 s interval. Thin lines represent all data at 3 s intervals.

**(B)** -CTT microtubules example kymographs and quantification of intensity over time.

**(i)** Example kymographs of GFP-katanin imaged using TIRF microscopy bound to control microtubules in the presence of 100 nM GFP-katanin (look-up table set to 0 – 9000). For each concentration, two kymographs are represented.

**(a)** Kymograph displays the total 5 minutes of imaging. The yellow dashed box is the 1-minute interval represented by the kymograph on the right (in ***b***). Horizontal scale bar is 5 µm; vertical scale bar is 1 min.

**(b)** Kymograph made from the same exact microtubule and imaging as the left kymograph, with all frames displayed taken at an exposure time of 0.06 s; the total length of time represented in the vertical direction is 1 min. The time of the right kymograph is the same minute outlined in the yellow dashed box on the left kymograph (in ***a***). The fluctuations in intensity are more obvious in the higher time resolution kymograph. Due to the size of this kymograph, it has been rescaled by 50% compared to the left kymograph. Horizontal scale bar is 5 µm; vertical scale bar is 3 s. The look up tables (LUT) for all kymographs is set to the same level of 0-9000 in a 32-bit image prior to conversion to 8-bit for displaying.

**(c)** Quantification of the intensity of the GFP-katanin on the microtubule over time. The intensity profile, averaged over 10 pixels along the microtubule length was measured over time using the low time resolution kymographs. Regions of interest on the microtubule (green line, microtubule) and off the microtubule (black line, background) were quantified and plotted over time.

**(ii)** Example kymographs of GFP-katanin imaged using TIRF microscopy bound to control microtubules in the presence of 5 nM GFP-katanin (look-up table set to 0 – 800). For each concentration, two kymographs are represented.

**(a)** Kymograph displays the total 5 minutes of imaging. For 100 nM, the intensity of the GFP-katanin is fairly constant and depolymerization is obvious as well as severing around 4 minutes. The yellow dashed box is the 1-minute interval represented by the kymograph on the right (in ***b***). Horizontal scale bar is 5 µm; vertical scale bar is 1 min.

**(b)** Kymograph made from the same exact microtubule and imaging as the left kymograph, with all frames displayed taken at an exposure time of 0.06 s; the total length of time represented in the vertical direction is 1 min. The time of the right kymograph is the same minute outlined in the yellow dashed box on the left kymograph (in ***a***). The fluctuations in intensity are more obvious in the higher time resolution kymograph. Due to the size of this kymograph, it has been rescaled by 50% compared to the left kymograph. Horizontal scale bar is 5 µm; vertical scale bar is 3 s. The look up tables (LUT) for all kymographs is set to the same level of 0-9000 in a 32-bit image prior to conversion to 8-bit for displaying.

**(c)** Quantification of the intensity of the GFP-katanin on the microtubule over time. The intensity profile, averaged over 10 pixels along the microtubule length was measured over time using the low time resolution kymographs. Regions of interest on the microtubule (green line, microtubule) and off the microtubule (black line, background) were quantified and plotted over time.

**(iii)** Quantification of the intensity normalized to the background for GFP-katanin binding along control microtubules over time. Data from parts **(i,c and ii,c)** are divided to give the signal (microtubule) to noise (background) for 100 nM (red line) and 5 nM (blue line). Thick lines represent averaged data over 30 s interval. Thin lines represent all data at 3 s intervals.

As described in the main text, the intensity of the GFP-katanin fades when bound to -CTT microtubules, but not when bound to control microtubules. The fading is due to photobleaching and takes place at a rate comparable to bleaching of the background fluorescence in the image. For control microtubules, the intensity stays high because the katanin is mobile and able to dissociate and re-associate from the bath. For -CTT microtubules, the fewer katanin molecules bind, but they are also less mobile and appear affixed to the filament, causing their intensity to photobleach.

At the lowest concentration of GFP-katanin, 5 nM, individual GFP-katanins are clearly shown to bind to, diffuse on the surface, and dissociate from control microtubules (Supp. Fig. 4Biib), as reported previously (1). Katanin molecules bind less frequently and are less dynamic on -CTT microtubules resulting in photobleaching.

The intensity over time, after normalization by the background, increased for 100 nM GFP-katanin, and then leveled off. This is similar to what we have reported previously (2). The 5 nM katanin on control microtubules appeared to be constant over time after normalizing by the background. This could be explained by two possibilities: 1. the GFP-katanin bound to the microtubule is photobleaching at the same rate as the background, or 2. the majority of the “signal” on the microtubule is due to the background intensity, which is photobleaching, and the individual molecules are not contributing to the signal much. When we examine the signal for -CTT microtubules, we find that the 100 nM katanin signal is fairly constant, but slightly decreasing over time. Since the katanin concentration was high for this data set, the data likely implied that the katanin is photobleaching like the background. The data set for the 5 nM had the most apparent loss of signal, even after correcting for the background photobleaching. This could imply that the molecules were dissociating over time in addition to bleaching. The rate of loss was not high, but appeared to be larger than the shot noise of the measurement (Supp. Fig. 4Biii).


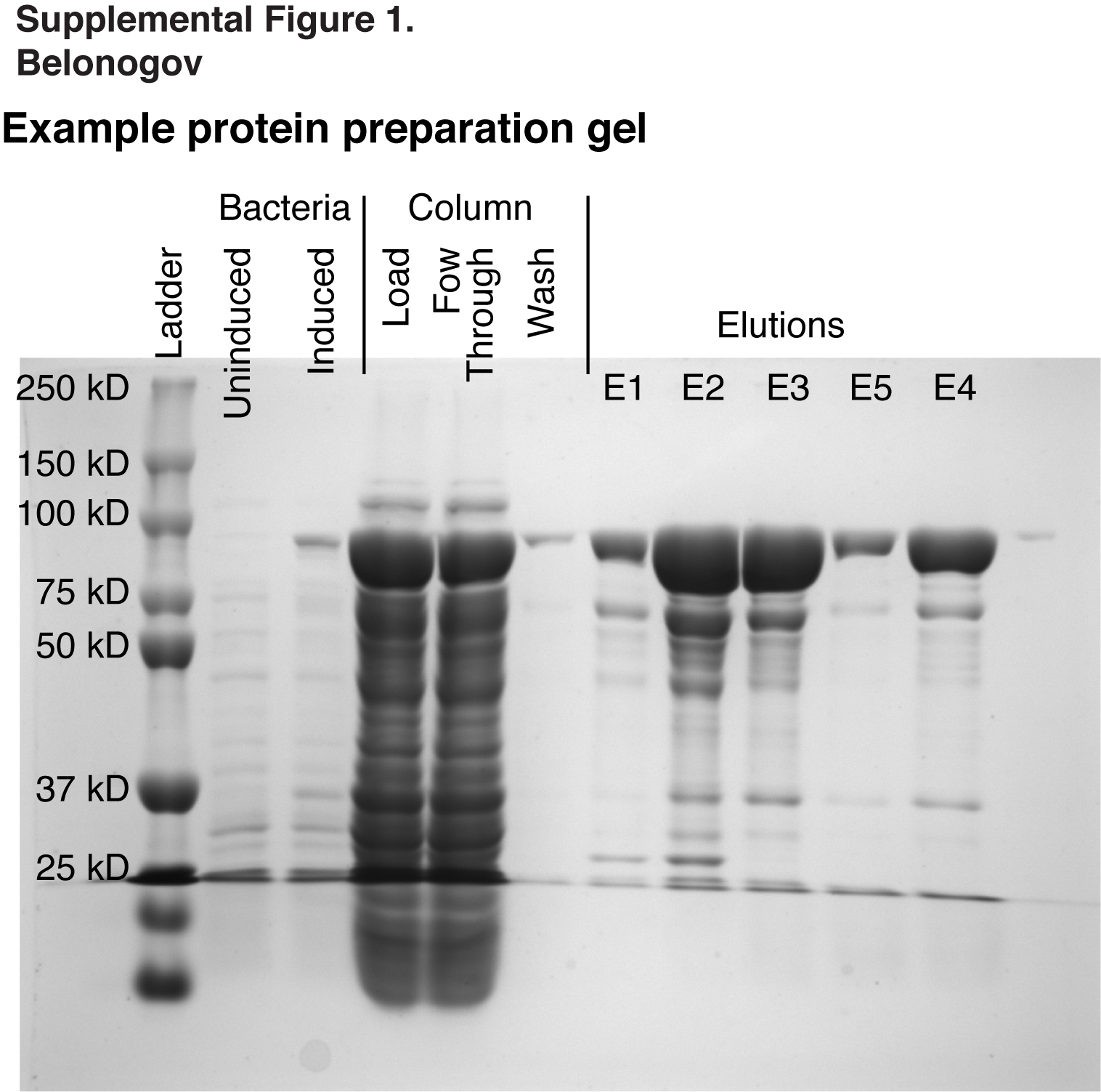


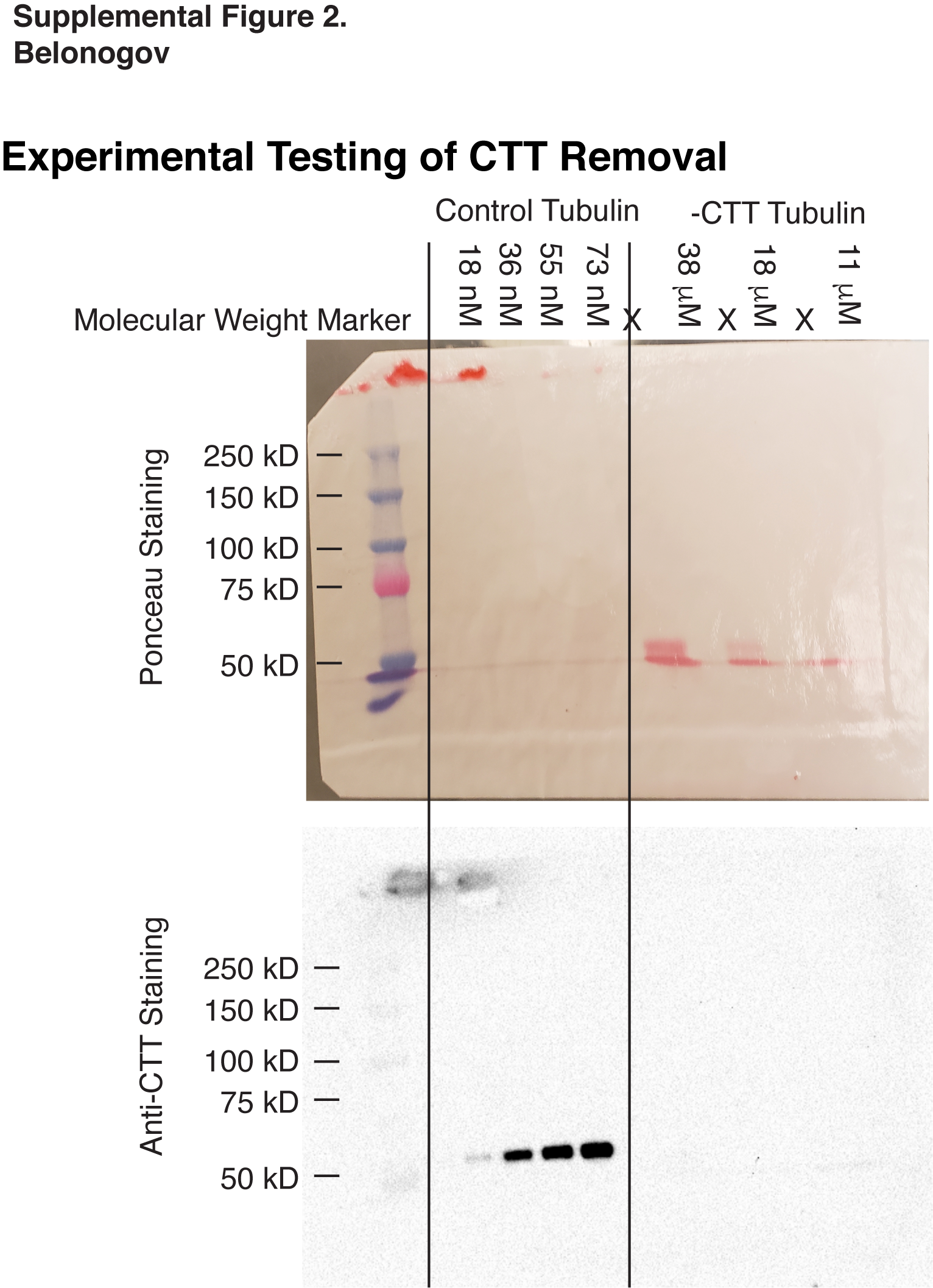


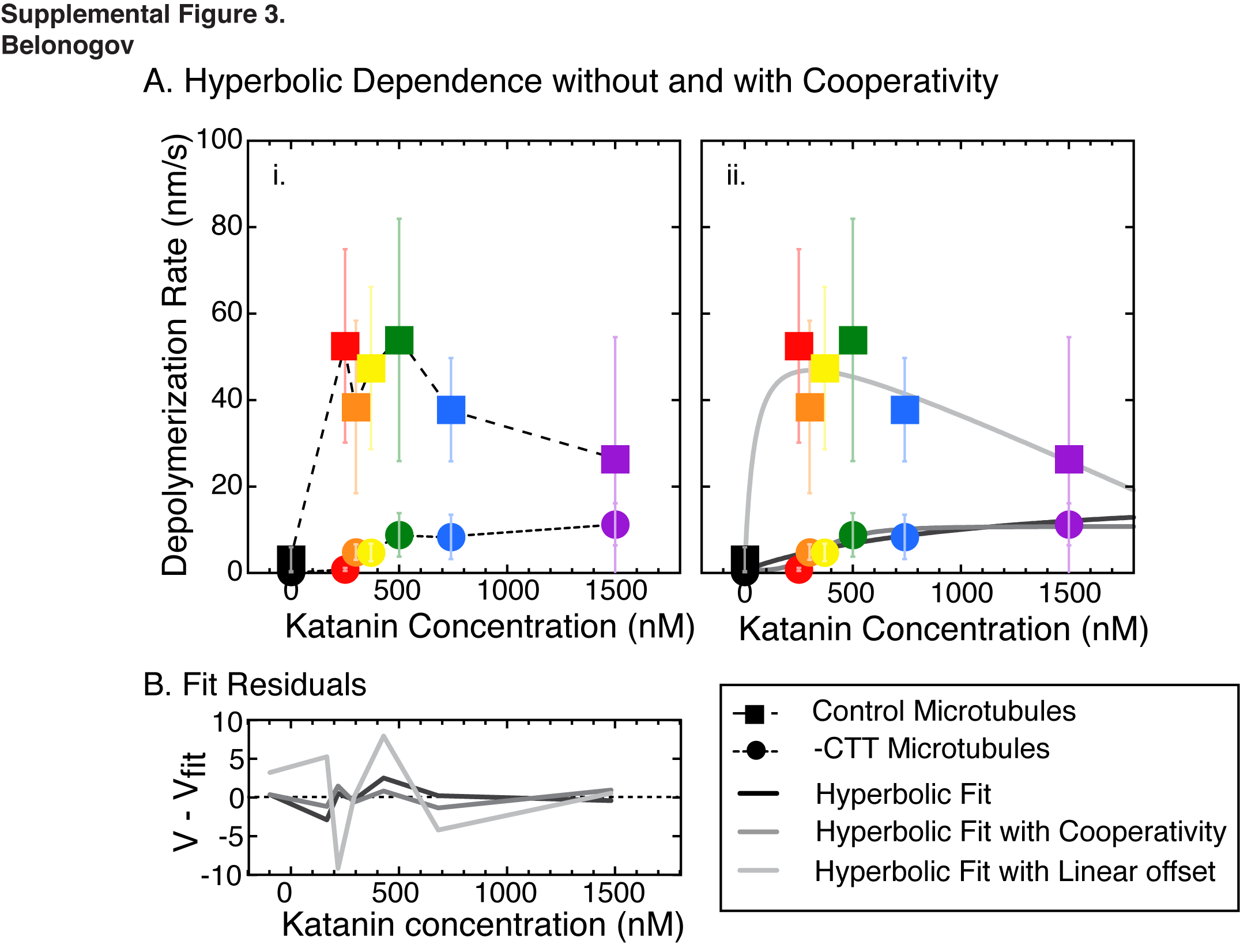

Supplement: Supplementary file 1 — Supplemental Table 1 Supporting Information [file CM-76-254-s001.docx]
